# The influence of perceived vocal traits on trusting behaviours in an economic game

**DOI:** 10.1177/17470218211010144

**Published:** 2021-04-17

**Authors:** Sarah Knight, Nadine Lavan, Ilaria Torre, Carolyn McGettigan

**Affiliations:** 1 Department of Psychology, University of York, York, UK; 2 Speech, Hearing and Phonetic Sciences, University College London, London, UK; 3 Department of Psychology, Royal Holloway, University of London, London, UK; 4 Division of Robotics, Perception and Learning, KTH Royal Institute of Technology, Stockholm, Sweden

**Keywords:** Voice, vocal traits, trust, vocal communication

## Abstract

When presented with voices, we make rapid, automatic judgements of social traits such as trustworthiness—and such judgements are highly consistent across listeners. However, it remains unclear whether voice-based first impressions actually influence behaviour towards a voice’s owner, and—if they do—whether and how they interact over time with the voice owner’s observed actions to further influence the listener’s behaviour. This study used an investment game paradigm to investigate (1) whether voices judged to differ in relevant social traits accrued different levels of investment and/or (2) whether first impressions of the voices interacted with the behaviour of their apparent owners to influence investments over time. Results show that participants were responding to their partner’s behaviour. Crucially, however, there were no effects of voice. These findings suggest that, at least under some conditions, social traits perceived from the voice alone may not influence trusting behaviours in the context of a virtual interaction.

## Introduction

When presented with human faces, viewers make snap judgements about the social traits of their owners, such as their trustworthiness (Todorov et al., 2009). These “first impressions” are made automatically and very rapidly (Willis & Todorov, 2006); they are also remarkably consistent across different viewers (Todorov et al., 2009). Similar first impression formation has been demonstrated for voices: participants readily make trait judgements about speakers after hearing only brief utterances, and such judgements are highly consistent across listeners (McAleer et al., 2014). However, the implications of these judgements for our behaviour towards others remain unclear. In particular, two critical questions must be answered. First, do voice-based first impressions actually influence behaviour towards the voice’s owner? Second, do voice-based first impressions interact over time with the voice owner’s observed actions, further influencing the listener’s behaviour? A small number of existing studies suggest that this may indeed be the case (Torre, 2017; Torre et al., 2015, 2016, 2020); however, the voice materials in these studies incorporated a number of different linguistic, social, and identity-related cues. The effects of voice quality alone on listener behaviour therefore remain unknown. In this study, we explored this question using an investment game paradigm.

### The investment game

In the investment game, participants decide how much money to invest in a partner; the investment is multiplied by a fixed factor (e.g., tripled) before being passed on to the partner, and the partner then chooses how much of this larger amount to return. The game therefore provides a means of measuring trust and—if it is iterated several times within the pair—tracking learning and reputation formation (Berg et al., 1995).

When using the investment game to examine face-based judgements, participants typically view their “partner’s face” before playing each round of the game. In these studies, though, the “partner” is usually a computer algorithm, with a pattern of behaviour pre-determined to be either generous or mean. Existing studies using this method suggest that faces judged as more trustworthy accrue higher initial investments (Chang et al., 2010; Rezlescu et al., 2012; van’t Wout & Sanfey, 2008). Other positive facial characteristics, such as attractiveness (Wilson & Eckel, 2006) and smiling (Scharlemann et al., 2001), also increase initial investments. This may be due to a “halo effect,” in which a generalised positive assessment of a person influences judgement of their individual attributes (Nisbett & Wilson, 1977); however, it may also be due to aspects of social signalling. For example, a smile may be interpreted as a willingness or invitation to cooperate (Scharlemann et al., 2001).

During iterated games, the effects of perceived facial traits are further modulated by experience of the partner’s actual behaviour. For example, Chang et al. (2010) found that participants not only invested more in partners who reciprocated frequently but also that first impressions and partner behaviour interacted, with partners who both initially appeared trustworthy and subsequently behaved in a trustworthy fashion prompting the largest investments overall. Wilson and Eckel (2006), meanwhile, report a “beauty penalty,” in which returns were lower for attractive (and hence apparently trustworthy) partners whose initial investments did not live up to participants’ expectations. Similar “beauty penalties” have been reported using different economic games (Andreoni & Petrie, 2008; Solnick & Schweitzer, 1999).

### Voice-based investment games

Drawing on face-based paradigms, Torre and colleagues explored the influence of voices during investment games (Torre, 2017; Torre et al., 2015, 2016, 2020). Their results suggest that the investment game might provide a fruitful means of investigating the social consequences of voice-based first impressions. In a series of studies, they demonstrated that investments were influenced by attributes of voices including regional accents, prosodic features such as fundamental frequency (f0), and emotional expressivity (“smiling voice”; Pickering et al., 2009); furthermore, some of these attributes interacted with the partner’s behaviour to further influence investments, and their relative influence changed over the course of the game. For example, they report a “beauty penalty,” in which trustworthy voices were “punished” with particularly low investments if the partner behaved meanly (Torre, 2017).

However, Torre et al.’s studies used linguistically complex speech stimuli featuring content relevant to the game (e.g., “I will return more money from this moment, this is a promise.”). In addition, several of their experiments used socially stereotyped regional accents (e.g., Liverpudlian English; see Bishop et al., 2005). As a result, it is possible that the observed effects of voice in these studies reflect the interplay of speaking style with the message being relayed and/or listeners’ prior beliefs or stereotypes about individuals from certain geographical areas. Consequently, it is unclear how first impressions generated from the sound of the voice itself might affect listener behaviour. To isolate the effects of voice quality, we therefore adopted two key methodological modifications to Torre et al.’s investment game paradigm. First, we used simple speech stimuli, comprising utterances of no more than five syllables in length (e.g., “Get ready, it’s me.”). Second, we used only a Standard Southern British English (SSBE) voice. Finally, using only one speaker, we also controlled for any idiosyncratic identity-related vocal cues which may have affected listener judgements. We thus report on an investment game study featuring two versions of the same voice that were judged to differ in relevant social traits but which contained minimal linguistic, identity-related, or socially stereotyped cues to such traits. The aim was to investigate whether, under these conditions, (1) voices judged to differ in relevant social traits accrue different initial investments and (2) first impressions of voices interact with the behaviour of the “partner” over time to influence participant investments in the longer term.

## Hypotheses

*H1*. Overall investments will be higher for generous partners than mean partners.
*H2*. There will be an interaction of voice × behaviour, such that investments are (1) highest overall for a partner who is both generous and also represented by a trustworthy voice and (2) lowest overall for a partner who is mean and yet represented by a trustworthy voice (akin to a “beauty penalty”).
*H3*. Initial investments will be higher for a more trustworthy voice than a less trustworthy voice.
*H4*. There will be an interaction of voice × behaviour × time, such that the relative importance of perceived vocal traits compared to the partner’s actual behaviour changes during the course of the game. This interaction will primarily indicate a gradual decrease in the weighting of vocal trait information over time (as found by Chang et al., 2010). However, it may additionally indicate the specific stage(s) in the game at which any “penalties” are applied.

## Materials

### Voice tokens

Recordings were made of 12 male speakers of SSBE (aged 21–41) . All recordings were made in sound-attenuated booths using desktop computers running Audacity (https://www.audacityteam.org/; RRID = SCR_007198) and with either a Røde NT1A microphone (Røde Microphones, Sydney, Australia) or a Neumann TLM103 microphone (Neumann, Berlin, Germany).

Speakers produced a selection of short, neutral phrases (e.g., “Hello” and “Get ready”). The chosen version of each phrase was extracted from the full recording and centred to remove DC drift, and all phrases were root-mean-square (RMS) amplitude normalised. The phrases were then concatenated with intervening silence to make five voice tokens as follows:

1. Hello, it’s me
2. Hello, get ready
3. Get ready, it’s me
4. It’s me, get ready
5. Right, get ready

#### Voice manipulation

The primary manipulation of interest was vocal trustworthiness. Some promising initial investigations notwithstanding (Belin et al., 2017; Ponsot et al., 2018), the precise acoustic correlates of trustworthiness are unclear (Knight et al., 2018). Furthermore, it was anticipated that speakers would lack an intuitive understanding of how to change their voices if asked to sound “more trustworthy.” However, there are other social traits whose vocal realisations are more intuitive and better understood, and which are closely linked to trustworthiness. As discussed above, facial displays of positive affect have been shown to be related to trustworthiness in face perception (Oosterhof & Todorov, 2009), and influence behaviour during investment games (Scharlemann et al., 2001). The voice tokens for the current studies were therefore directly manipulated in terms of their perceived positive affect with the anticipation that these manipulations would also result in differences in perceived trustworthiness.

All speakers recorded both positive (i.e., “cheerful”) and neutral versions of each phrase, from which positive and neutral versions of each of the five voice tokens were created. An initial pilot study (*N* = 24) was carried out online in which participants were asked to rate the voice tokens for happiness and trustworthiness on 7-point Likert-type scales. The data from this pilot were used to select one speaker for use in the main study. The ratings (Table 1) indicated that this speaker’s positive voice tokens were perceived as significantly happier and significantly more trustworthy than the same speaker’s neutral voice tokens (both *p* < .0001; Fisher’s Exact Test).

**Table 1.** Average happiness and trustworthiness ratings (mean [*SD*]) obtained in the initial pilot for the voice tokens used in the main study.

Condition	Average trustworthiness rating	Average happiness rating
Positive	4.73 (1.13)	4.56 (1.17)
Neutral	3.58 (1.55)	2.45 (1.06)

*SD*: standard deviation.

This initial pilot involved participants listening to all 12 original speakers in both positive (cheerful) and neutral conditions. However, during the investment game itself, participants only ever heard one voice in one condition (see section “Procedure” below). We therefore carried out a second pilot study in which each participant heard a concatenated string of the five voice tokens from one condition only: that is, each participant only ever heard one voice in one condition, as in the investment game itself. Twenty four participants were assigned to each voice condition and rated the voice for trustworthiness only, again on a 7-point scale. None of these participants had participated in any earlier piloting or in the main investment game studies. Average trustworthiness ratings for the chosen speaker from this second pilot are given in Table 2. These results confirm that the positive voice was perceived as significantly more trustworthy than the neutral voice even when heard in isolation (*p* < .001; Fisher’s Exact Test). For simplicity, we therefore refer hereafter to the positive voice as “trustworthy” and to the neutral voice as “less trustworthy.” A summary of the acoustic characteristics of the voice tokens produced by the chosen speaker is given in Table 3.

**Table 2.** Average trustworthiness ratings (mean [*SD*]) obtained in the second pilot for the voice tokens used in the main study.

Condition	Average trustworthiness rating
Positive	5.29 (1.33)
Neutral	4.17 (0.96)

*SD*: standard deviation.

**Table 3.** Acoustic characteristics (mean [*SD*]) of the voice tokens used in the main study (averaged across all five voice tokens for each condition).

Condition	F0 mean (Hz)	F0 min (Hz)	F0 max (Hz)	Duration (s)
Positive	160.00 (6.31)	90.38 (15.78)	206.10 (6.68)	1.44 (0.14)
Neutral	116.58 (1.18)	98.88 (0.12)	130.82 (1.30)	1.34 (0.09)

*SD*: standard deviation.

## Procedure

### The investment game

**Figure 1.**
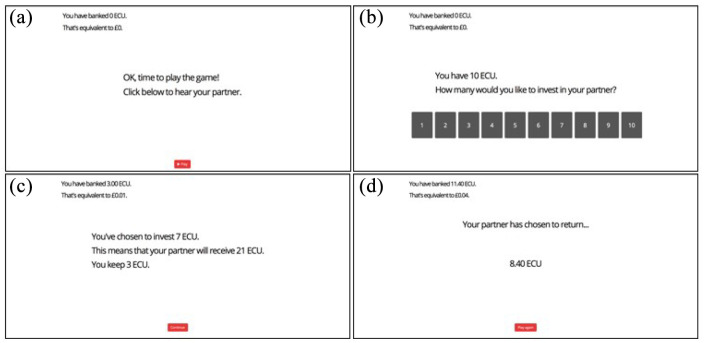
The structure of a single round (trial) of the investment game. Top left (a): the participant is invited to play a new round of the game, and clicks to hear their partner’s voice. Top right (b): having heard the partner’s voice, the participant selects how many ECU (experimental currency units) to invest in their partner. Bottom left (c): the participant is given a reminder of their choice and of the implications for their partner. Bottom right (d): the participant finds out how much the partner has chosen to return. A “bank” is always present in the top left of the screen; this updates dynamically to reflect the participant’s earnings (in both ECU and GBP).

Participants were informed that they were going to play a game with a partner during which they could earn a bonus payment; that is, they were offered a monetary incentive which they believed would be directly related to their success in the game. In reality, all participants received a fixed bonus at the end of the game, reflecting the maximum amount they could have won (£1.20). The investment game was played using “ECU” (experimental currency units), with an exchange rate of 3,000 ECU to 10 pence (GBP). On each trial (“round”), participants were given 10 ECU and had to decide how much to invest in their partner, with a minimum investment of 1 ECU. This initial investment was tripled and sent to the “partner,” who then “chose” how much to return. The returned value was always a certain proportion of the initial investment. Generous partners returned 120%, 150%, 180%, 210%, or 240% of the initial investment with equal probability, and mean partners returned 0%, 30%, 60%, 90%, and 120% of the initial investment with equal probability. Within each behaviour condition (generous/mean), each of the five possible return values occurred the same number of times during the course of the game. A “bank” on the screen kept the running total of participants’ earnings (in both ECU and GBP). On each round, participants first heard one of the five voice tokens before making their investment. The “partner” was the same throughout the game (i.e., participants only ever heard voice tokens from one voice in one condition), and each of the five voice tokens occurred the same number of times during the course of the game. The order of voice tokens and return values was fully randomised within each participant. Figure 1 illustrates the structure of one round of the game.

Before starting the main game, participants watched a short animation demonstrating how a round of the game might work. They then played five practice rounds. These practice rounds were identical in structure to those in the main investment game, but without any voices. Instead of hearing a voice token, participants saw a speech bubble containing text (“Hi! Get ready to play!”). The return values used during these practice trials (70%, 80%, 100%, 110%, and 130%) gave participants experience of receiving returns that were both less and more than their initial investment.

### Online data collection

All data, including pilot data, were collected online. Participants were recruited using the recruitment platform Prolific (www.prolific.co), and data were collected using the testing platform Gorilla (www.gorilla.sc; Anwyl-Irvine et al., 2020). Participants were reimbursed in line with Prolific’s recommended rates (at least £5/hr, not including the bonus payment) and provided informed consent before being allowed to proceed to the studies. For all studies, participants completed a headphone screening task (taken from Woods et al., 2017) to ensure they were wearing headphones and listening in a suitably quiet environment.

### Sampling

The only comparable studies to date are those by Torre et al. (2015, 2016, 2020; Torre, 2017). In those studies, the authors were able to detect main effects and interactions in linear mixed models using sample sizes in the order of 20 participants per condition. In this study, data were therefore collected from 80 participants. Since the study had a 2 (voice) × 2 (behaviour) design, this ensured that data were collected for 20 participants per condition.

### Analyses

All data were analysed using the following packages (and functions) in R Version 3.5.1: *stats* (*fisher.test, lm*); *lme4* (*lmer*); *lmerTest; lsmeans; MuMIn; car; HLMdiag; BayesFactor* (*ttestBF, lmBF*).

### Ethical approval

The initial pilot and main study were approved by the College Ethics Committee at Royal Holloway, University of London (approval no. 928). The second pilot was approved by the local ethics officer at the Department of Speech, Hearing and Phonetic Sciences at University College London (approval no. SHaPS-2019-CM-030).

## Main study

### Participants

Eighty participants (42 female) took part in the study. All were aged between 18 and 40 (average = 28.0; *SD* = 6.5) , spoke fluent English, described their nationality as United Kingdom, had no reported hearing difficulties, and had an approval rate of more than 75% on Prolific. None of the participants had taken part in any other studies associated with this project. Participants were randomly assigned to one of four conditions (2 voice [trustworthy/less trustworthy] × 2 behaviour [generous/mean]) and played 20 rounds of the game.

### Results

#### Overall investments

Overall investments for each condition are shown in Table 4. A linear regression was run with voice (trustworthy/less trustworthy) and behaviour (generous/mean) as categorical predictor variables and each participant’s average investment as the outcome variable. Visual inspection of standardised residuals and a non-significant result from Levene’s test for homogeneity of variance indicated that no model assumptions had been violated. A main effect of behaviour indicated that average investments were higher overall for generous partners than mean partners, *F*(1, 76) = 85.21, *p* < .001; adjusted *R*^2^ = .51. This supports H1. There was no effect of voice, and no interaction of voice × behaviour. This does not support H2. Furthermore, a Bayesian analysis comparing the full model to a model containing only behaviour as a predictor produced a Bayes factor of 0.096. Working on the basis that a Bayes factor < 0.33 represents evidence in favour of the null hypothesis (Dienes, 2014), this provides strong evidence against any influence of the voice on overall investments.

**Table 4.** Average overall investments (mean [*SD*]) for each of the four conditions.

Behaviour	Voice	Average investment in ECU
Generous	Trustworthy	7.68 (2.51)
Generous	Less trustworthy	7.61 (2.36)
Mean	Trustworthy	4.00 (2.64)
Mean	Less trustworthy	4.46 (2.97)

*SD*: standard deviation; ECU: experimental currency units.

#### Initial investments

To investigate the effect of the partner’s voice on initial investments, data were analysed from Trial 1 only. Investments were pooled across behaviour conditions (because participants had not yet received any returns, so no distinction between generous and mean partners had yet been made).

Average initial investments in the trustworthy and less trustworthy voices were 6.15 (*SD* = 2.28) and 5.70 (*SD* = 2.02), respectively. Since one-off investments are categorical in nature, a non-parametric test was used for analysis. Fisher’s Exact Test indicated no significant difference in investments between the trustworthy voice and less trustworthy voice (*p* = .37). This does not support H3. A Bayesian analysis comparing an effect of voice to the null hypothesis produced a Bayes factor of 0.340. This value is between 0.33 and 3, and as such is inconclusive: it cannot be taken as evidence for the null hypothesis, nor can it can be taken as evidence for H3. It is therefore the case that H3 remains unsupported.

#### Interactions between voice and behaviour over time

**Figure 2.**
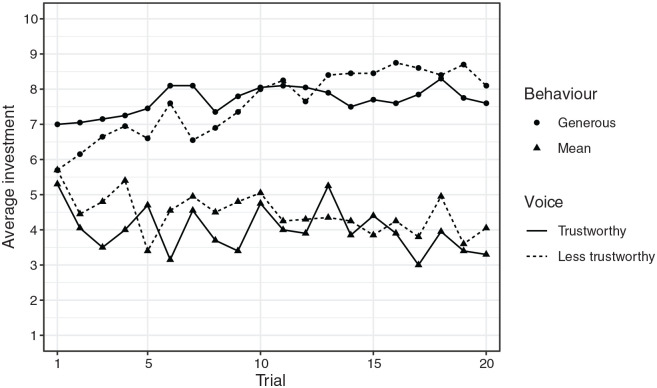
Average investments on each trial across all four conditions.

Figure 2 shows average investments for each condition on a trial-by-trial basis. To examine the relative effect of partner’s voice and behaviour over time, participants’ investments were averaged across four consecutive bins of trials. For each participant, investments were averaged across Trials 2–5, Trials 6–10, Trials 11–15, and Trials 16–20. Trials were binned in this way to assess behaviour change over time while not overfitting to fluctuations arising from a specific local combination of return values.

A linear mixed effects model was run with voice, behaviour, and bin number as categorical predictor variables, binned investments as the outcome variable, and participants as random intercepts. A backwards stepwise procedure was used to obtain the most parsimonious model.^1^ The final model included a main effect of behaviour and an interaction of behaviour × bin, *F*(1, 80) = 91.95, *p* < .001, *F*(3, 240) = 6.28, *p* < .001; marginal *R*^2^ = 0.45. Post hoc tests (pairwise comparisons, Tukey corrected) indicated that investments were significantly lower in the first bin than in the final two bins, but only in the generous condition; in the mean condition, there were no significant differences in investments between any of the bins. The final model did not contain a main effect of voice, and no interactions between voice and either behaviour or bin. This does not support H4. Furthermore, a Bayesian analysis comparing the full model to the final model produced a Bayes factor of 0.016. This provides extremely strong evidence against any influence of the voice on investments during the course of the game.

## Discussion

In this study, we explored the influence of perceived vocal traits on investments during an economic game played with a virtual partner. The results provide support for H1 (see “Hypotheses” above): overall investments were significantly higher for generous partners than mean partners. This is in line with findings from the investment game more generally and confirms that participants were playing the game as expected. However, the results did not provide support for any of the remaining hypotheses. There was no interaction between voice and behaviour (H2); initial investments were not significantly higher for the more trustworthy voice than the less trustworthy voice (H3); and there were no interactions between voice, behaviour, and time (H4). Furthermore, Bayesian analyses showed that results typically provided strong evidence in favour of the null hypotheses for H2–H4: in others words, the data speak strongly against an effect of a partner’s perceived vocal traits on investments, either initially, overall, or at different stages of the game.

Our results are in direct contrast to the literature exploring the effect of perceived facial traits on investments, in which facial trustworthiness has been shown to affect both initial investments and overall investments, and also to interact with the partner’s behaviour over time (Chang et al., 2010; Rezlescu et al., 2012; Scharlemann et al., 2001; van’t Wout & Sanfey, 2008; Wilson & Eckel, 2006). The results are also in contrast to those obtained by Torre and colleagues (2015, 2016, 2020; Torre, 2017), which suggested that vocal attributes contributing to perceived trustworthiness—such as prestige accents and expression of positive affect—influence participant investments during voice-based investment games, and in some cases interact with the speaker’s behaviour.

The within-speaker approach of this study allowed us to create significantly differing impressions of trustworthiness across the two voice conditions while controlling for other vocal attributes such as accent, sex, age, and specific talker identity. However, it is possible that the absolute ratings of the two voice conditions were not extreme enough to produce trait inferences of sufficient strength to influence behaviour. We obtained mean high versus low trustworthiness differences of 1.15 and 1.12 (first and second pilot studies, respectively), with absolute means around the centre of the 7-point rating scale. In the face literature, the precise ratings given to faces used as “trustworthy” or “untrustworthy” exemplars are often not specified; however, the work of van’t Wout and Sanfey (2008) may be instructive. They report ratings of faces obtained after participants had played the investment game: “Mean subjective trustworthiness rating of faces of partners after the game was 4.1 (*SD* = 1.65, range 1–7).” (p. 799). Using ±1 standard deviation, we can infer a range of ratings between around 2.45 and 5.75; a difference of 3.30, which far exceeds that found here. Indeed, the results of Belin et al. (2017) suggest that it simply may not be possible to generate wide-ranging percepts of trustworthiness in simple utterances through talker-generated manipulations of affect. In their study, the authors created a continuum of vocal trustworthiness by averaging sets of naturally produced low- or high-trustworthiness voices and then morphing in equal steps between the two resulting low- and high-trustworthiness prototypes. The continuum also included “caricatures” at its extremes, which morphed *beyond* the prototypes. Trustworthiness ratings across the entire continuum, including these caricatures, ranged from 189 to 307 on a scale of 0–500 (a difference of 118), while ratings of the voices within the range of the prototypes ranged from 211 to 279 (a difference of 68). Relative to the scales used, this latter range is even smaller than the range of ratings obtained for the voice tokens used in our study, suggesting a generalised difficulty in generating divergent percepts of vocal trustworthiness, and particularly so without the use of artificial manipulation.

Torre and colleagues (2015, 2016, 2017, 2020) previously reported that “smiling” voices received higher overall investments in iterated investment games, using multiple talkers and accents. The aim of this study was to control for accent and isolate the effects of voice quality alone; we therefore used an SSBE-accented speaker, as this is considered to be the standard form of spoken English in the United Kingdom. Here, we found no effect of positive versus neutral speaking styles on implicit trusting behaviours in the investment game. Torre et al.’s findings suggest it is possible that implicit trust might vary across accents and talker identities, perhaps via the additional/interactive engagement of broader social stereotypes (e.g., SSBE being perceived as the “prestige” accent; a lisp being perceived as “posh”). Those previous studies did not report on the relationship between explicit ratings and implicit trusting behaviours. However, crucially our study shows that manipulations of voice quality that yield significant differences in explicit ratings of trust are in fact not sufficient to generate different profiles of implicit trust in all cases. This has implications for the use of explicit ratings as a proxy for how listeners behave towards a voice, and bears relevance to applied settings such as the selection of voice identities for use in public announcements, or to advertise commercial products.

Another potential contributor to the contrast between our findings and those of Torre et al. may be the linguistic content of the stimulus sets. This study was designed to examine the effects of voice quality on listeners’ trusting behaviours. Thus, we used short phrases whose semantic content was compatible with the iterated task (e.g., “Get ready, it’s me”) but that were otherwise unrelated to the outcomes of each round of the game. In contrast, the voice stimuli used in Torre et al.’s studies featured relatively long utterances that dealt directly with the game and the concept of trust (e.g., “You have to trust me. I have every intention to repay your trust.”). These stimuli therefore introduced not just additional linguistic complexity but also the possibility for participants to feel either reassured or deliberately deceived (depending on the value of the following return) by the voice. It may be that a higher level of task relevance in the content of speech is necessary for participants to learn about their partner as well as about the pattern of round-by-round investment outcomes (as found here).

## Conclusion

It is already known that listeners can make complex social trait judgements from voices after hearing only brief utterances. However, results from this study suggest that controlled variations in intrinsic voice qualities without concomitant linguistic manipulations may have only limited influence on listeners’ trusting behaviour. It seems plausible that previous findings of voice/speaker effects on listener trust behaviours have arisen from complex interactions between idiosyncratic vocal traits, linguistic content, and social stereotypes. More work is needed to unpick this complex network of potential influences on participant behaviour during voice-based investment games.

## Notes

1. This procedure was implemented through manual checking and effect removal, as follows: the most complex possible model (i.e., full factorial: all main effects and all possible interactions) was run first. Non-significant effects were then removed. Effect removal took place one level at a time, as follows: if the highest-level interaction was a three-way interaction and was not significant, it was removed and the model re-run. Each non-significant two-way interaction was then removed in turn, and the model re-run, and so on. Non-significance was determined using Wald χ^2^ tests to compare a model to a nested version of the same model with the critical term removed. Where multiple effects at the same level were non-significant, the first term to be removed was that which resulted in the largest drop in the model AIC value. The principle of marginality was observed: that is, if a higher-level interaction was included in the model, then the nested lower-level interactions (“marginal effects”) were also retained. For example, if A*B*C was kept in the model, then the model also included A*B, A*C and B*C. For the final model, QQ plots of model residuals and random effects were used to visually assess whether assumptions of normality of residuals had been violated.
